# PseAAC-General: Fast Building Various Modes of General Form of Chou’s Pseudo-Amino Acid Composition for Large-Scale Protein Datasets

**DOI:** 10.3390/ijms15033495

**Published:** 2014-02-26

**Authors:** Pufeng Du, Shuwang Gu, Yasen Jiao

**Affiliations:** 1 School of Computer Science and Technology, Tianjin University, Tianjin 300072, China; E-Mails: shuwanggu@gmail.com (S.G.); yasenjiao@gmail.com (Y.J.); 2 Tianjin Key Laboratory of Cognitive Computing and Application, Tianjin University, Tianjin 300072, China; 3 Department of Computer Science, City University of Hong Kong, Kowloon, Hong Kong

**Keywords:** general form, large-scale datasets, pseudo-amino acid composition

## Abstract

The general form pseudo-amino acid composition (PseAAC) has been widely used to represent protein sequences in predicting protein structural and functional attributes. We developed the program PseAAC-General to generate various different modes of Chou’s general PseAAC, such as the gene ontology mode, the functional domain mode, and the sequential evolution mode. This program allows the users to define their own desired modes. In every mode, 544 physicochemical properties of the amino acids are available for choosing. The computing efficiency is at least 100 times that of existing programs, which makes it able to facilitate the extensive studies on proteins and peptides. The PseAAC-General is freely available via SourceForge. It runs on both Linux and Windows.

## 1. Introduction

Over the last few years, machine learning has been introduced to predict protein structures and functions. In these studies, one of the keys is to formulate the protein sequences with a mathematical form that can reflect the intrinsic correlation with their structures and functions. To be more specific, this mathematical form should keep representing a protein sequence with a discrete form yet without completely losing its sequence-order information. The pseudo-amino acid compositions (PseAAC), which was originally introduced to predict protein attributes [1], is a typical mathematical form in this regard.

Ever since its first appearance, the PseAAC formulation has been widely applied for studying various problems in protein science, such as predicting eukaryotes and prokaryotes protein subcellular locations [2–11], protein sub-subcellular locations [12–22], membrane protein subcellular locations [23–26], viral protein subcellular locations [27,28], protein structural classes [29–35], secondary structures [36], super-secondary structures [37], quaternary structural attributes [38,39], GPCR classes [40–42], enzyme families [43,44], membrane protein types [45–47], metalloproteinase families [48], risk types of human papillomavirus [49], cell-wall lytic enzymes [50], cyclic proteins [51], allergenic proteins [52], bioluminescent proteins [53], DNA-binding proteins [54], GABA(A) receptor proteins [55], bacterial virulent proteins [56], essential proteins [57], anti-cancer peptides [58], anti-bacterial peptides [59], protein-protein interactions [60], protein solubility [61], drug-target network [62], and many more [63–76]. Recently, it was applied to represent DNA sequences in identifying the recombination spot [77].

Many different types of information, such as gene ontology annotations, functional domain compositions, and sequential evolution information, have been integrated skillfully with the concept of PseAAC to represent protein samples in order to enhance the prediction quality of their attributes. In essence, the protein sample thus formulated were actually various modes of Chou’s general form PseAAC, as clearly indicated by Equations 9–14 in a comprehensive review [78]. On the contrary, the Type I PseAAC [1] and Type II PseAAC [79] belong to Chou’s special form PseAAC. The modes of Chou’s special form PseAAC can be calculated by several programs, such as PseAAC server [80], PseAAC-Builder [81] and the propy package [82].

However, so far no publicly accessible program could calculate Chou’s general PseAAC. The current PseAAC-General is a universal software platform for users to generate various modes of general form PseAAC, including several widely used modes, such as the gene ontology mode [3], functional domain mode [83], and sequential evolution mode [18]. It is anticipated that PseAAC-General will become a very useful tool in bioinformatics, computational proteomics, and system biology.

## 2. Results and Discussion

The current PseAAC-General can generate 13 different modes of general form PseAAC, including conventional amino acid composition, di-peptide composition, tri-peptide composition, Type I PseAAC, Type II PseAAC, the gene ontology mode, the functional domain mode, the sequential evolution mode, the normalized Moreau-Broto autocorrelation coefficients, the Moran autocorrelation coefficients, the Geary autocorrelation coefficients, the composition-transition-distribution (CTD) descriptors and the quasi-sequence order descriptors. In every mode, 544 types of physicochemical properties are available for choosing. Over 20,000 different descriptor values can be calculated.

We list several commonly used modes of general form PseAAC as well as some program features in PseAAC-General program in Table 1. Several modes are uniquely available in PseAAC-General, which include the gene ontology mode, the functional domain mode and the sequential evolution mode. These modes have been mentioned in existing programs [81,82]. However, no program implemented these modes.

PseAAC-General provided two methods for the users to create their own desired modes. The first method is called the Binary Extension Module (BEM). The gene ontology mode and functional domain mode were actually implemented by this method. A set of tools was provided along with the PseAAC-General, so that the users can create their own BEM to represent all kinds of descriptive information, which includes but not limited to the gene ontology annotations and the functional domain compositions.

The other method is the Lua script module. Lua script language is a very simple programming language that has been considered in analyzing sequence annotations [90]. We provided a programming interface that allows the user to use Lua script to access the internal data structures and functions of PseAAC-General. Furthermore, the algorithm modules of PseAAC-General can be replaced by the user-defined Lua script modules. This provides the maximal flexibility for the user-defined mode. Actually, the sequential evolution mode was implemented in this way.

**Figure 1.**
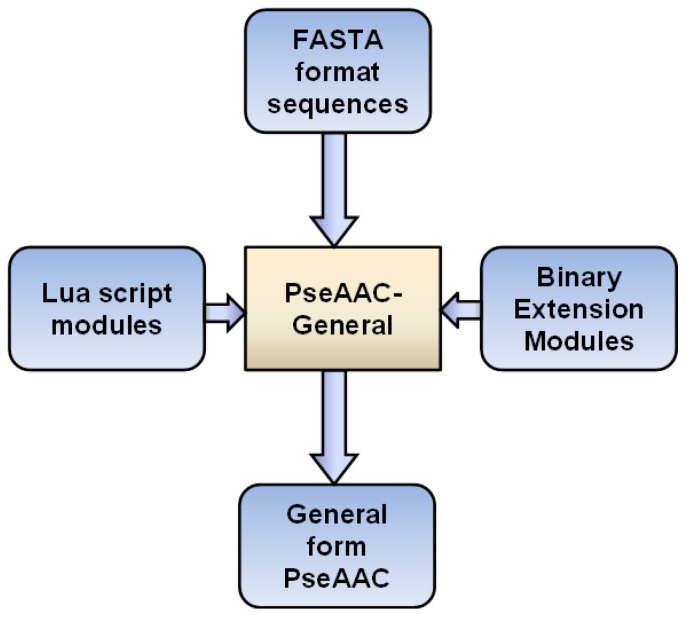
The data flow of pseudo-amino acid composition (PseAAC)-General. The input data is FASTA format sequences. The output data is general form PseAAC. The mode of the general form PseAAC is chosen by the users. For the modes, which are implemented by Binary Extension Modules or Lua script modules, the corresponding modules should be loaded as well.

Because of these extension modules, the input to the PseAAC-General is not only the protein sequences. These extension modules should also be loaded if they are needed. We illustrate the data flow of PseAAC-General in Figure 1.

The usefulness of PseAAC-General is undisputed. In the early days of general form PseAAC, every study had to implement the PseAAC independently. This may bring a number of problems, including but not limited to inconsistent results, different computation efficiency and different basis in comparing predictive performance. PseAAC-General can serve as a standard program that saves time for all these studies. Furthermore, our program eliminates those unforeseen problems that were brought by the different implementations of PseAAC.

PseAAC-General is much faster than existing programs. We tested PseAAC-General by using it to calculate Type I PseAAC with default parameters. On the same machine that we tested PseAAC-Builder [81], it can process about 17,000 sequences per second. This is about 100 times faster than PseAAC-Builder. In other words, PseAAC-General can convert the entire Swiss-Prot database to Type I PseAAC within 30 s, while PseAAC-Builder needs about 40 min.

## 3. Implementations

PseAAC-General is released under GNU GPL (GNU General Public License). It can be integrated with other programs in the source code level. We have ported PseAAC-General to both Linux and Windows platforms. A GUI (Graphical User Interface) module was provided for both platforms. The users, who do not familiar with the command line, can use PseAAC-General through GUI. However, it should be noted that the most efficient way is the command line, which was designed to follow the GNU command line standard.

PseAAC-General was designed to be a stand-alone program running on the local machine without internet connection requirements. Therefore, we did not include the online sequence retrieving function within the program. On the other hand, the propy package has perfectly implemented the retrieving function. The best choice for the users is to let PseAAC-General work side by side with the propy package. For example, the users can use Propy to retrieve protein sequences and call PseAAC-General to calculate the PseAAC, as python environment has the built-in ability to call external programs, like PseAAC-General. In future versions of PseAAC-General, a similar function will be implemented. PseAAC-General and all its extension modules can be downloaded from its website [91]. To facilitate further studies, all source code of PseAAC-General, including the main program, GUI module and all extension modules, can be freely downloaded from the SourceForge website [92]. We also provided detailed documents within the software package, so that the users can learn not only how to use the existing modes, but also how to create their own modes by building their own extension modules. For the users’ convenience to test their own modes, we provided four different testing dataset with different size. These testing datasets can also be downloaded from the website. Along with the testing datasets, we provided simple testing scripts to demonstrate the usage of PseAAC-General in a common case. The users can simply try the testing scripts to learn how to use the program.

Because the gene ontology mode and the functional domain mode should be upgraded along with the Swiss-Prot database, we deployed a cloud-computation based server in Amazon EC2 (Elastic Cloud 2, Amazon.com Inc., Seattle, WA, USA) to automatically upgrade the relevant extension modules on monthly basis.

## 4. Conclusions

As PseAAC-General is a very powerful and very flexible computation tool, we believe that PseAAC-General will facilitate all studies that apply the general form PseAAC, including those existing modes and those modes in development.

However, as a final reminder, we would like to remind the users to read the manual of PseAAC-General and those literatures describing the algorithm of general form PseAAC carefully before using it. Because of the powerful function and the flexibility of PseAAC-General, using it in your study without knowing the algorithms and technics behind the program and the source code could be very risky.

## Figures and Tables

**Table 1.** Comparison of program features.

Program Functions a	PseAAC-General	PseAAC-Builder	Propy	PseAAC Server
Physicochemical Properties	544	544	8	6

Output Features				

Type I PseAAC [1]	Y	Y	Y	Y
Type II PseAAC [79]	Y	Y	Y	Y
Amino acid composition	Y	Y	Y	Y
di-Peptide composition	Y	Y	Y	Y
tri-Peptide composition	Y	N	Y	N
Normalized Moreau-Broto autocorrelation [84,85]	Y	N	Y	N
Moran autocorrelation [86]	Y	N	Y	N
Geary autocorrelation [87]	Y	N	Y	N
Composition-Transition-Distribution (CTD) [88]	Y	N	Y	N
Quasi-sequence order [89]	Y	N	Y	N
Gene ontology mode [83]	Y	N	N	N
Functional domain mode [83]	Y	N	N	N
Sequential evolution mode [18]	Y	N	N	N
Other functions				
User defined	Y	N	N	N
Online updates	Y	N	N	N
Graphical User Interface (GUI)	Y	Y	N	Y
Execution efficiency b	~17,000 seqs/s	~170 seqs/s	N.A.	~15 seqs/s

a The program functions that were compared. There are three groups of functions, including the physicochemical properties, the sequence features that can be generated and the other function properties of the software. Y = YES; N = NO;

b the execution time for PseAAC-General and PseAAC-Builder was tested on a dataset containing over 510,000 sequences by the wall-clock time. The execution time for PseAAC-Server was tested on a dataset containing 500 sequences due to the limitation of the service and the internet connection conditions. The execution time for Propy was not tested due the limitation of testing environments. Seqs/s means sequences per second.
